# Toxoplasmosis—Awareness and Knowledge of Pregnant Women in Rural Areas of Malakand Region, Pakistan

**DOI:** 10.1155/2023/4603066

**Published:** 2023-05-13

**Authors:** Wali Khan, Hafeez ur Rahman, Yousef Abdal Jalil Fadladdin, Naseem Rafiq, Robi Naz, Patricio R. De los Rios-Escalante, Shabir Ahmad, Shouaa Abdulaziz Alrobaish, Noorah Saleh Al-Sowayan

**Affiliations:** ^1^Department of Zoology, University of Malakand, Lower Dir, Pakistan; ^2^Department of Zoology, Abdul Wali Khan University, Mardan, Pakistan; ^3^King Abdulaziz University, Faculty of Sciences, Department of Biological Sciences, Jeddah, Saudi Arabia; ^4^Universidad Catolica de Temuco, Facultad de Recursos Naturales, Departmento de Ciancias Biologicas Quimicas Casella 15-D, Temuco, Chile; ^5^Núcleo de Estudios Ambientales UC Temuco, Casilla, Temuco, Chile; ^6^Department of Zoology, Hazara University, Mansehra, Pakistan; ^7^Department of Biology, College of Science, Qassim University, Buraidah, Saudi Arabia

## Abstract

**Background:**

The current study was carried out between October 2017 and October 2018 to explore knowledge, attitudes, practices, and information sources regarding toxoplasmosis among pregnant women in Malakand region, the northwestern part of Pakistan. The current study was carried out between October 2017 and October 2018.

**Methods:**

A structured questionnaire was used to interview the women after taking verbal informed consent. Graphpad version 5 was used to indicate the differences. Significant was considered as a *P*-value of less than 0.05. This study revealed poor knowledge regarding toxoplasmosis.

**Results:**

Overall, 31.2% of the respondents showed good knowledge, and 39.2% showed moderate knowledge. On the other hand, 29.5% of the participants showed poor knowledge about toxoplasmosis. The average knowledge score of pregnant women was 79 ± 12.2, which is considered to be within the scale of good knowledge. Number of children within the pregnant multipara women was significantly associated with knowledge about toxoplasmosis. Pregnant women who measured in number of childbirths within a women showed the highest mean score of 42.3 ± 13.3 with 57 (44.8%) displaying a good knowledge level. Pregnant women with more than one child had significantly higher (<0.0001) knowledge scores compared to women with one child or none child. The majority of pregnant women with one child used the social media, followed by mass media as sources of information about toxoplasmosis. Scientific sources of information were used more commonly by pregnant women with none of the child birth.

**Conclusion:**

Pregnant women knowledge regarding toxoplasmosis was poor as compared to attitudes and practices. Health workers and newspapers/magazines were the main sources of information.

## 1. Introduction

Toxoplasmosis is a worldwide disease with no geographical boundaries [1]. *Toxoplasma gondii* is an intracellular protozoan parasite that affects a wide range of intermediate hosts, including all warm-blooded animals and humans [2]. The domestic cat is one of the most important hosts for oocyst development [3].

The accidental ingestion of spore bearing oocysts and consumption of raw meat are the principal, and blood transfusion as well as organ transplant are the secondary sources of infection. Unpasteurized milk from infected goats, on the other hand, is a major source of toxoplasmosis, especially in children. The placenta is considered one of the important routes of transmission of toxoplasmosis from mother to offering in pregnancy [4]. In areas where toxoplasmosis is common, the congenital toxoplasmosis is found [5]. Close contact with oocysts, contaminated cat feces, and soils are some of the ways this parasite disease is transmitted [6].

Because of the transmission of IgG antibodies from mother to child and the lack of particular symptoms in neonates, diagnosing neonatal infections can be problematic. Diagnosis of *T. gondii* infection is commonly established by serological testing for immunoglobulin M (IgM) and immunoglobulin G (IgG) antibodies [7]. IgM antibodies are produced in the first week after infection and decline to undetectable levels within months. IgG antibodies appear 2–3 weeks after IgM antibodies and indicate recent infection [8]. However, the detection of IgG and IgM antibodies is still the most commonly used approach for primary screening [9]. Prevention depends on the prevalence, risk of infection, laboratory facilities, and financial resources [10]. Because most women are infected during their reproductive years, the chance of prenatal infection is considerable.

Knowledge, attitude, and practices studies on toxoplasmosis have shown that most of the women at childbearing age had a limited knowledge of methods to prevent toxoplasmosis [11]. Toxoplasmosis might be asymptomatic in the later months of pregnancy or appear weeks or months after delivery in pregnant women. However, it has the potential to cause abortion and stillbirth [12]. Toxoplasmosis is a serious problem in high-prevalence locations, and studies have suggested effective preventive approaches [13]. The research conducted by [14] shows 40.6% prevalence overall. The current study's objective was to assess pregnant women's knowledge, attitudes, and practices about toxoplasmosis in rural Malakand, Pakistan.

## 2. Materials and Methods

### 2.1. Study Area

The current study was carried out on the pregnant women of Malakand region, Pakistan, between October 2017 and October 2018 to know knowledge, attitude, practices, and sources of information of pregnant women towards toxoplasmosis. This region is situated 35° 29′ 59.99″ N and 72° 00′ 0.00″ E with 1420 m elevation. This is a mountainous region with high peaks in the north reaching up to a height of 6000 m above sea level, and the height decreases slowly from north towards south along the river Swat. Malakand region is situated in the temperate zone where winters are cold with temperature reaching below freezing point, while summers are hot and humid due to heavy monsoon rains, and the temperature may reach up to 32°C [15].

### 2.2. Data Collection and Sampling

A structured questionnaire was designed and translated into local language (Pashto). The questionnaire consisted of socio-demographic questions (age, education, number of children, gestational age, and history of miscarriage/abortion), knowledge about toxoplasmosis, attitudes, and practices (complication of pregnant women, controlling responsibility, and source of information). Students were trained in interviewing techniques, and filled in the questionnaire during their visits to the concerned hospitals.

### 2.3. Statistical Analysis

Collected data were analyzed using the Graphpad version 5, the statistical software. A statistically significant association between variables is considered to exist if *P* < 0.05.

## 3. Results

The present survey reports knowledge, attitude, practices, and information sources of 237 pregnant women on toxoplasmosis in rural areas of Malakand, the northwestern parts of Khyber Pakhtunkhwa, Pakistan. Very rare information is available on the prevalence of *T. gondii* infection in pregnant women in this part of Pakistan.

### 3.1. Socio-Demographic Characteristics of the Respondents

Most of the women 88.1% had the age greater than 18 years and 71.7% were illiterate (71.7%). Majority of the pregnant women (53.8%) had number of children >1 followed by women without children (28.6%) and 10.1% of the women were having only one child. The pregnant women in second trimester were in majority (56.1%) followed by (24.4%) in third trimester, while only 19.4% were in first trimester. About 62% had miscarriages due to infection with toxoplasmosis (Table 1).

Toxoplasmosis was well-understood by nearly a third of the participants (31.2%). The percentage of pregnant women who had a moderate understanding of toxoplasmosis was 39.2%. Only 29.5% of the participants had a poor understanding of toxoplasmosis (Table 1). Women under the age of 18 years had better knowledge (39.2%) than women over the age of 18 (30.1%). Similarly, women under the age of 18 had higher moderate knowledge (42.8%) than women over the age of 18 (38.7%). Women under the age of 18 had a lower rate of knowledge (17.8%) than women over the age of 18 (31.1%). The relationship between knowledge categories and ages was found to be insignificant (*P* = 0.32).

In terms of reading and writing ability, illiterate women have more good knowledge (32.3%) than literate women (28.3%), although literate women have more moderate knowledge (47.7%) than illiterate women (35.8%). However, literate women have less low knowledge (23.8%) than illiterate women (31.7%). The relationship between knowledge categories and ability to read and write was found to be insignificant (*P* = 0.22).

In terms of the number of children a pregnant woman has, women who have more than one child have better knowledge (44.9%) than women who have only one child (25%) or none at all (12.7%). Women with no children had the most moderate (53.4%), followed by women with one child (33.3%), and women with more than one child (44.8%), (30.7%). Women with one child (41.6%) had the poorest knowledge, followed by women without a child (33.7%), while women with more than one child (24.4%) had the least. There was a strong association between knowledge categories and the number of children (*P* = 0.0001).

In terms of gestational age, women in their third trimester had greater good knowledge (34.4%) than those in their second (31.5%) and first trimesters (26%). Women in the first and third trimesters had higher (41.3%) moderate knowledge than women in the second trimester (37.5%). The women had the less knowledge, coming in third (24.1%), second (30.8%), and first scores (32.6%), respectively. There was no significant link between knowledge categories and gestational age (*P* = 0.8097).

In terms of miscarriage or abortion, women who have never had a miscarriage or abortion have a good knowledge level (36.6%) than women who have had a miscarriage or abortion (27.8%). Women who have had a miscarriage or abortion, on the other hand, have a moderate knowledge level (40.8%) than women who have never had a miscarriage or abortion (36.6%). Women who have never had a miscarriage or abortion (26.6%) had low knowledge than women who have had a miscarriage or abortion (31.2%). The association between knowledge categories and the history of miscarriage or abortion was found to be negligible (*P* = 0.3631).

### 3.2. Knowledge about Toxoplasmosis

**Figure 1:**
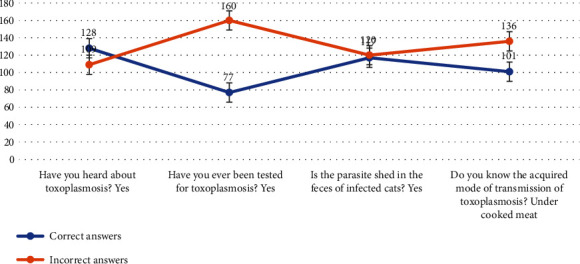
Knowledge of pregnant women towards toxoplasmosis of Malakand region, the northwestern part of Pakistan (*n* = 237).

Most of the women, 54.1%, indicated they had ever heard of toxoplasmosis while 45.9% were not known. Most of the respondents (67.5%) were of the view that they have tested as compared to 32.4% who have not ever tested for toxoplasmosis. When asked about shedding of the parasite in the cat's feces, 49.3% replied yes while 50.6% stated no. In response to the question “Do they know about the acquired mode of transmission” 42.6% answered for undercooked meat while 57.4% don't know (Figure 1).

### 3.3. Attitudes and Practices Related to Toxoplasmosis

In terms of attitudes and practices, 63.7% of pregnant women answered that they do not have major difficulties following infection with *T. gondii,* but the remaining women believed that toxoplasmosis can cause serious complications. When asked if an unborn or newborn kid can have major difficulties after contracting toxoplasmosis, she said yes. Majority of the women (79.3%) were of the view that the newborn children do not develop severe problems after toxoplasmosis, while only 20.6% were voted for complication with toxoplasmosis. Most of the respondents (77%) do not give correct answer in response to the question that toxoplasmosis can be prevented by avoiding eating undercook meat, while only 23% of the respondents known that toxoplasmosis can be prevented by avoiding eating undercook meat. In response to the question who is the responsible body to control toxoplasmosis? (Community awareness) 15.6% were known that community involvement is the right way to control; however, 84.4% of the participants were of the view that community involvement is not the prior for controlling toxoplasmosis (Table 2).

### 3.4. Information Sources

The most popular source of information for toxoplasmosis in pregnant women was social media (37.9%), followed by mass media (34.1%) and science-based information (27.8%). When it came to information sources, the majority of respondents under the age of 18 were more likely to use them than those above the age of 18. There was no significant link between the source of information and age groups (*P* value is 0.9710). Literate people were discovered to utilize said information sources more than illiterate people when it came to their abilities to read and write (*P* value is 0.041). Women with more than one child used mass media the most (40.9%), women with one child used social media the most (50%), and women with no children used science-based information sources the least (43%) (*P* value is 0.002). In connection to gestational age, women in the third trimester were found to use mass media and social media extensively to obtain knowledge regarding toxoplasmosis, whereas women in the first trimester were found to utilize science-based information extensively (*P* value is 0.005).

Pregnant women with a history of miscarriage/abortion were found to use mass media extensively, whereas those without a history of miscarriage/abortion relied heavily on social media and science-based information (*P* value is 0.005) (Table 3).

## 4. Discussion

Research on toxoplasmosis conducted in different parts of the world revealed that both males and females were found variably infected. Females were highly infected as compared to male [16], and on the other hand, males were more infected than females [17, 18].

Age wise prevalence was noted as 39.4% in people of the age group 25–34 years as compared to other age groups [16]. Similar age groups were infected with 33.3% prevalence reported from Nigeria [19]. A low prevalence rate of 8.18% was observed in the age group of 5–14 years, which is less than 23.1% reported from Iran [17]. The infection rate was low in the age group 35–44 years, which has also been reported from other laboratories [18, 19]. It has also been found that *T. gondii* infection increased with age, lower educational level, populous living conditions, and soil-related occupations [20, 21].

Toxoplasmosis can be controlled in pregnant women by adopting a number of factors including washing of raw fruits and vegetables before eating [22].

Women's health education is very imperative to avoid maternal toxoplasmosis. Women's education is needed regarding their eating habits and antenatal care. Eating raw and undercooked meat should be avoided [23]. People should be educated to reduce the number of cats in their houses, which sheds oocysts in the surroundings and thus help in reduction of environmental contamination of oocysts of *T. gondii* [24]. Toxoplasmosis was more common in people between the ages of 20 and 40. *T. gondii* infection was only seen in uneducated pregnant women. Only 1.32% of the patients studied were positive for IgM and IgG antibodies, according to a study conducted by Khan et al. [25].

According to Andiappan et al. [26], the knowledge, attitude, and practices of pregnant women about toxoplasmosis are lower than expected. They have also been noted the reasons that majority of the pregnant women in rural areas lead pastoral life, no education, no access to media, and no focusing on the disease. The diagnostic facilities in health centers are the main constraints of the disease. The present study revealed that most of the pregnant women (67.5%) had never been experienced for infection with *T. gondii*. In this contest, current investigation is comparable to the study conducted by Amin et al. [27] who reported 58 (14.5%) of the pregnant women who were tested for toxoplasmosis during pregnancy but they do not have any idea about the type of test. The reports published in some parts of the world showing relationship between drinking raw milk and human infection with toxoplasmosis [28, 29].

## 5. Conclusion

Current study revealed that majority of the pregnant women were not aware about toxoplasmosis, its preventive measures, risk factors, symptoms, and timing. However, attitudes and practices were better and this is due to their local knowledge that makes them sure to prevent all types of infection. Educating the local people can play an important role in preventing toxoplasmosis. There must be an urgent need for medical professionals to receive refresher training, as well as for diagnostic facilities to be strengthened and for pregnant women to be advised to check for toxoplasma infection on a frequent basis.

## Figures and Tables

**Table 1:** Socio-demographic characteristics of pregnant women of Malakand region, the northwestern part of Pakistan (*n* = 237).

Demographic variable		*N*	Knowledge category (%)			Knowledge score	*X* ^2^	*P* value
			Poor	Moderate	Good			
Overall		237	70 (29.5)	93 (39.2)	74 (31.2)	79 ± 12.2		
Age (years)	<18	28	5 (17.8)	12 (42.8)	11 (39.2)	9.33 ± 3.7	2.233	0.3274
	>18	209	65 (31.1)	81 (38.7)	63 (30.1)	69.6 ± 9.86		
Ability to read and write	Literate	67	16 (23.8)	32 (47.7)	19 (28.3)	22.3 ± 8.50	2.985	0.2248
	Illiterate	170	54 (31.7)	61 (35.8)	55 (32.3)	56.6 ± 3.78		
Number of children	1	24	10 (41.6)	8 (33.3)	6 (25)	8 ± 2	27.10	<0.0001
	>1	127	31 (24.4)	39 (30.7)	57 (44.8)	42.3 ± 13.3		
	None	86	29 (33.7)	46 (53.4)	11 (12.7)	28.6 ± 17.5		
Gestational age (trimester)	First	46	15 (32.6)	19 (41.3)	12 (26.0)	15.3 ± 3.51	1.595	0.8097
	Second	133	41 (30.8)	50 (37.5)	42 (31.5)	44.3 ± 4.93		
	Third	58	14 (24.1)	24 (41.3)	20 (34.4)	19.3 ± 5.03		
History of miscarriage/abortion	Yes	147	46 (31.2)	60 (40.8)	41 (27.8)	49 ± 9.84	2.026	0.3631
	No	90	24 (26.6)	33 (36.6)	33 (36.6)	30 ± 5.19		

**Table 2:** Attitude and practices of the pregnant woman about toxoplasmosis in Malakand region, the northwestern part of Pakistan (*n* = 237).

Questions (correct answer)	Correct answers		Incorrect answers	
Can *T. gondii* infection cause major difficulties in pregnant women? Yes	86	36.2	151	63.7
Can *T. gondii* infection cause major difficulties in prenatal and newborn children? Yes	49	20.6	188	79.3
Preventive measure for toxoplasmosis (do not eat undercook meat)	55	23.0	182	77.0
Who is the responsible body to control toxoplasmosis? (community awareness)	37	15.6	200	84.4

**Table 3:** Sources of information regarding toxoplasmosis among respondents in Malakand region, Pakistan (*n* = 237).

Demographic variable		*N*	Mass media (%)	Social media	Science-based information	*X* ^2^	*P* value
			*N* (%)	*N* (%)	*N* (%)		
Overall		237	81 (34.1)	90 (37.9)	66 (27.8%)		
Age (years)	<18	28	9 (32.1)	11 (39.2)	8 (28.5)	0.05878	0.9710
	>18	209	72 (34.4)	79 (37.7)	58 (27.7)		
Ability to read and write	Literate	67	22 (32.8)	19 (28.3)	26 (38.8)	6.351	0.0418
	Illiterate	170	59 (34.7)	71 (41.7)	40 (23.5)		
Number of children	1	24	8 (33.3)	12 (50)	4 (16.6)	16.97	0.0020
	>1	127	52 (40.9)	50 (39.3)	25 (19.6)		
	None	86	21 (24.4)	28 (32.5)	37 (43.0)		
Gestational age (trimester)	First	46	15 (32.6)	12 (26.0)	19 (41.3)	7.244	0.1235
	Second	133	45 (33.8)	52 (39.0)	36 (27.0)		
	Third	58	21 (36.2)	26 (44.8)	11 (18.9)		
History of miscarriage/abortion	Yes	147	69 (46.9)	55 (37.4)	39 (26.5)	15.50	0.0004
	No	90	12 (13.3)	35 (38.8)	27 (30)		

## Data Availability

The datasets generated and/or analyzed during the current study are not publicly available but are available from the corresponding author on reasonable request.
